# Description and preliminary experience with Virtual Visit Assessment (ViVA) during the COVID-19 pandemic, a structured virtual management protocol for patients with multiple sclerosis

**DOI:** 10.1007/s10072-021-05371-3

**Published:** 2021-06-15

**Authors:** Roberto Bergamaschi, Livio Tronconi, Daniele Bosone, Antonella Mastretti, Laura Jommi, Marco Andrè Bassano, Renato Turrini, Sara Benati, Marco Volpe, Jean Marie Franzini, Silvia Allodi, Giulia Mallucci

**Affiliations:** 1 Multiple Sclerosis Center, IRCCS Mondino Foundation, via Mondino 2, 27100 Pavia, Italy; 2 Novartis Farma S.P.A, Origgio, VA Italy; 3 Bip Life Sciences, Milan, Italy

**Keywords:** Multiple sclerosis, Management, COVID-19, Telemedicine, Protocol

## Abstract

In people with multiple sclerosis (PwMS), strict follow-up is essential. Telemedicine has the potential to overcome many of the difficulties in routine management. Herein, we present a structured protocol that can be used to remotely manage patients with MS, describing in detail the steps to be taken and exams needed at each stage. A working group was established which developed a tailored protocol that can be adapted to a variety of settings. The overall protocol consisted of 5 phases: enrolment, document sharing phase, pre-evaluation, virtual visit, and post-visit phase, which was divided into 14 individual steps. As of October 2020, 25 virtual visits have been carried out, all via Skype. The patient’s caregiver was present during visits and had an active role. The average duration of the virtual visit was 24 min, and that of the pre-visit and post-visit were around 15 min each. Overall satisfaction as rated by physicians was considered high (8.0 ± 0.5). Using the system usability scale (SUS), patients also favorably rated the virtual visit (96.6 ± 6.1). In 20% of cases, the virtual visit was not sufficient to provide adequate information and an in-person clinical visit was recommended. The described protocol has the potential to provide benefits for the healthcare system as well as patients and their caregivers both during and beyond COVID-19 pandemic.

**Supplementary Information:**

The online version contains supplementary material available at 10.1007/s10072-021-05371-3.

## Introduction

Multiple sclerosis (MS) is a chronic autoimmune disease, and is the most disabling neurological condition of non-traumatic etiology in young adults [1, 2]. In recent years, the incidence and prevalence of MS has been increasing although the reasons for this increase remain unclear [3]. In terms of etiological factors, MS is a complex disease and both genetic and environmental factors have been implicated. While a few genes have been reported to increase individual susceptibility to develop MS, and some environmental factors have been identified such as low levels of serum vitamin D and low UV light exposure, smoking, obesity, and infections have also been associated with the disease [4–6].

Early therapy is held to be the most effective method to limit permanent damage to the central nervous system. In this regard, reducing the time to achieve diagnosis of MS has become a priority for those treating the disease, also in light of the fact that there may be an early window of opportunity where effective therapy can significantly slow disease progression [7]. Indeed, with this concept in mind, the recently proposed revisions to the McDonald diagnostic criteria for MS have placed emphasis on early diagnosis and misdiagnosis, with the goal of prompt therapy [8]. With the availability of numerous disease-modifying therapies with different mechanisms of action and specialty care interventions, management of MS has become increasingly complex [9].

In MS patients, follow-up visits are essential in order to (i) follow the clinical evolution of the disease; (ii) monitor clinical effects, tolerability, and occurrence of adverse events related to therapy; (iii) detect the onset of symptoms related to the course of the disease at an early stage and recommend examinations or modify therapy accordingly; (iv) program interventions to minimize personal, social, and work problems, involving, if necessary, the family or a caregiver, specific professionals, and local services [10, 11]. Given the increased number of patients with MS, complexity of management, frequent need for follow-up visits, and limited availability of outpatient visits in some settings, there is thus an objective need for innovative management models. This is especially true in light of the current COVID-19 pandemic and its impact on reducing outpatient services capacity, given new safety procedures (e.g., distancing, sanification, PPE-personal protection equipment use), layout redesign, reallocation of clinical staff to emergency activities, patients no-show in follow-up visit induced by the risk of contagion in the hospital setting [12]. In this regard, telemedicine may be of benefit in overcoming some of the difficulties in management of MS patients.

Telemedicine is broadly considered diagnosis and treatment of patients remotely using digital communications [13, 14]. In many fields of medicine, telemedicine is an increasingly used means of patient management [13]. Several types of telecommunications have been suggested to be useful in management of patients with MS, including internet-based teleconferencing, store and forward technology, and home telemedicine using mobile communications. Moreover, it is considered to be a valid and cost-effective means of delivering specialist care for patients with MS and other neurological disorders [15, 16].

Despite its potential benefits, at present telemedicine is not widely used to manage patients with MS. This may be in part because its optimal implementation has not been described and, moreover, barriers still remain to its more widespread adoption, such as lack of compliance and low levels of patient engagement [15–18]. In addition, while there are many reports of the use of telemedicine, there remains limited evidence to date of its efficacy compared to standard in-clinic visits. Given the above, there is an undisputable need for increased adoption of remote solutions in neurology, even if teleconsultation cannot immediately replace “most” face-to-face interactions in the MS clinic. While digital connections do have massive potential, they still lack the essential finesse of human interaction. We advocate for a complementary hybrid model (teleconsultation can be helpful, for example, for routine follow-up visits, discussion of investigations, and address drug tolerability/compliance issues). This would therefore combine accessibility with reliability in a context of professionalism and evidence-based practice.

With the broad aim of increasing the use and overcome some of the barriers faced by telemedicine, we present a structured protocol that can be used to remotely manage patients with MS, describing the precise steps to be taken and exams needed at every stage of follow-up. Moreover, preliminary results on the efficacy of the protocol are also presented.

## Materials and methods

The structured protocol was initially developed by BIP Life Sciences with Novartis Farma support and the Multiple Sclerosis Research Centre, C. Mondino National Neurological Institute, Pavia, Italy, was identified as the pilot center.

A local working group was established which was composed of the Chief Hospital Administrator, Health Director, Health Presidium Director, and Executive Committee Director, as well as the respective heads of the MS clinic, legal office, and IT services. The virtual model for monitoring MS patients proposed herein was developed based on the following considerations. Measurement and sizing of patient flows by type of outpatient activity were estimated and routine patient flow was carefully analyzed to detect potential critical points. Next, the management processes of neurological visits were broken down to identify specific activities, players, roles, and support tools used in daily practice. The available technological solutions for remote physician–patient communication were then assessed to determine their suitability considering the above. Lastly, the challenges related to the introduction of a virtual visit were evaluated and tested on MS patients who needed neurological follow-up but who could not go to the MS Center during the COVID-19 pandemic. Physicians rated their satisfaction with the virtual visit by replying to an ad hoc questionnaire (Online Appendix 1). Patient experience was also assessed through the system usability scale (SUS) [19], a simple, 10-item attitude scale giving a global view of subjective assessments of the usability of a technical system. For each item, the user expresses his/her level of agreement (5 options from “Strongly disagree” to “Strongly agree”). Finally, the question for the patient “Would you recommend this system to another patient?” was assessed by a visual analog scale (VAS—range 0–10).

## Results

### Development of Virtual Visit Assessment

The overall project consisted of 5 main processes: (i) development of an organizational model; (ii) identification of the technology to be adopted; (iii) analysis of legal aspects; (iv) design of support tools; (v) assess the efficacy of the model.

In developing the organizational model, we considered the type of visit along with pre-visit activities, duration of the visit, and actions needed post-visit. The different players were identified (clinician, nurse, residents, patient, caregiver). In the protocol developed, sections were included on patient eligibility, exams and supportive tools, and practical organizational aspects. In terms of the technology adopted, IT services were contacted to identify the ideal platform to adopt for each structure and when feasibility trial was carried out. In this particular case, an existing platform already in use at the hospital (Healthmeeting) was adopted to record the virtual visits carried out using Skype. Information was given to patients and caregivers on the digital solution chosen and various legal aspects. Support tools were then identified and adapted to the digital solution chosen. All this information was used to develop a clinical support kit for participating centers and a starter kit for patients containing all relevant information, along with the parameters to be evaluated to assess the efficacy of the proposed model.

### Description of the protocol

**Fig. 1:**
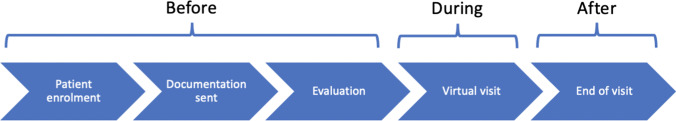
Phases of the virtual visit

**Fig. 2:**
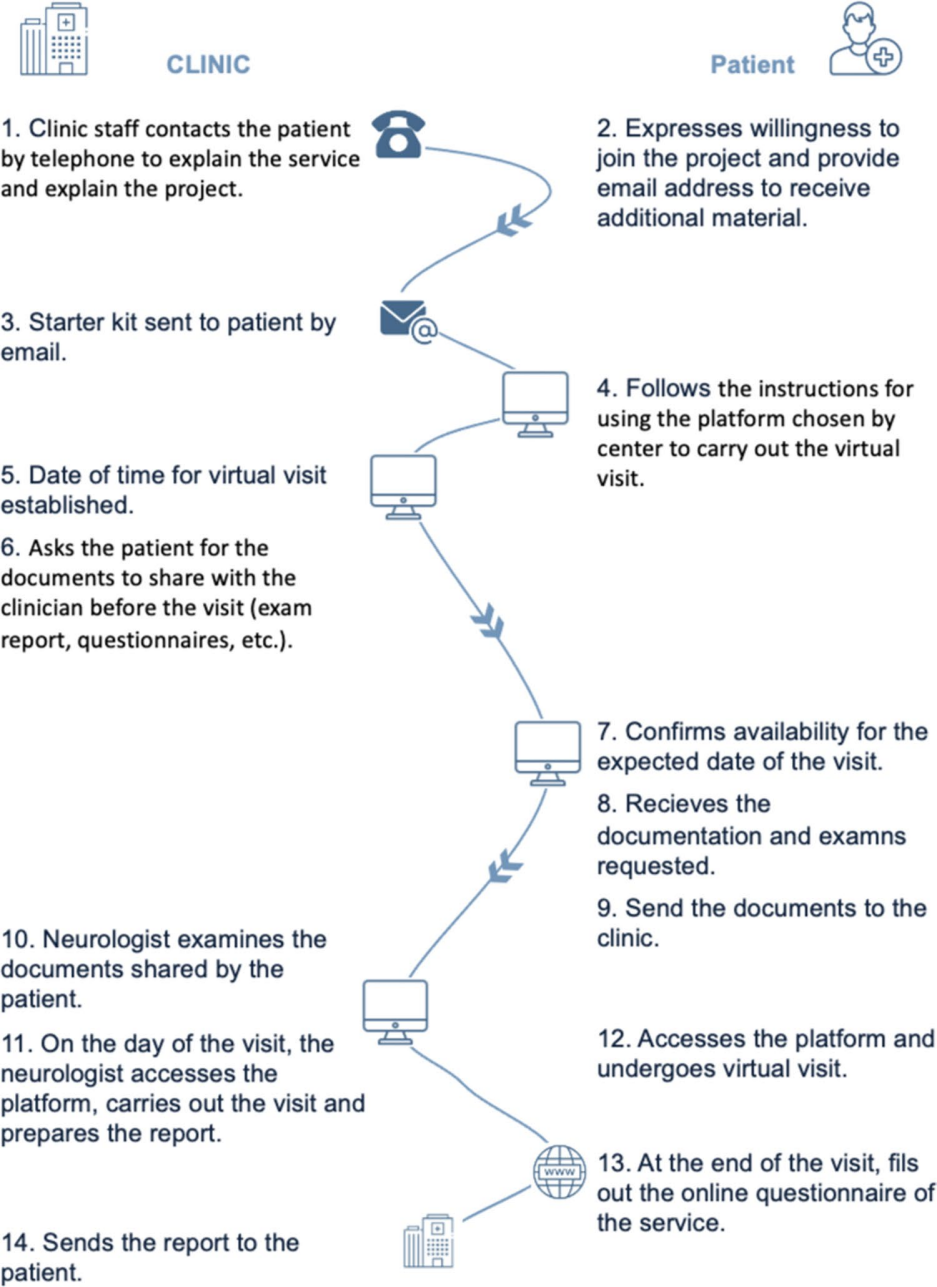
Summary of the overall protocol for the virtual visit

The phases of the virtual visit are shown in Fig. 1. These include an enrolment phase, document sharing phase, pre-evaluation phase, the virtual visit itself, and the post-visit phase. The overall protocol structure is shown in Fig. 2. The protocol for the virtual visit was divided into 14 steps to be sequentially carried between the MS center and patient. The virtual visit is initiated by the center’s office who contact the patient by telephone and explain the process. The patient then expressed his/her desire to participate, and if affirmed, a starter kit is sent by email. The patient then accessed the platform and followed the instructions that are utilized by the individual center. Next, the center’s office schedules a visit and informs the patient of the exams and documentation needed for the virtual visit. The patient then confirms their availability for the scheduled visit, obtains/performs the documentation/exams requested, and sends these to the center online. In the next step, a neurologist examines the documents sent prior to the virtual visit. The neurologist then carries out the virtual visit according to the checklist in the protocol, after which both the patient and neurologists compiled a satisfaction questionnaire. Finally, the neurologist sends the patient the report of the virtual examination.

The information requested before the virtual visit is detailed in Table 1 along with the structure of the visit itself. Prior to the visit, the patient must provide information regarding any instrumental and laboratory exams that have been requested along with information regarding pain, fatigue, etc., if required. During the virtual visit, the neurologist collects any relevant clinical history and evaluates the patient’s physical status, language, etc. similarly as one normally would during a standard in clinic visit. The therapy is confirmed or changed as needed, and any additional exams requested. The clinical report is then compiled and sent to the patient after the visit via the online platform.

**Table 1:** Structure of the virtual visit

Pre-visit
Patient • Undergo diagnostic exams (laboratory exams, MRI) • Other tests requested by the clinician - Pain - Spasticity - Fatigue - Depression - Physical tests (with video sent to clinic) • Share exams and tests with clinician
Physician • Evaluation of test results • Examine laboratory exams and MRI images
During virtual visit
Patient and physician • Identification of the patient • Collect clinical data • Comment in exam results (if needed) • Assessment of: - Physical functioning - Language - Gestures - Memory and attention • Answer patient and caregiver questions • Provide information on the disease • Provide information on overall management • Confirm therapy • Request new exams • Compile report
After virtual visit
Physician • Share report • Establish date of next follow-up visit

The visit could be tailored to the individual patient according to the physician’s judgement

A number of tests and exams were standardized in the protocol to evaluate the patient as shown in Table 2. These included optional questionnaires to evaluate pain, spasticity, depression, and fatigue, along with laboratory and instrumental exams, evaluation of physical status with standard tests, and assessment of the patient’s dialog. As mentioned, the virtual visit concludes with any future exams needed, confirmation or changes to therapy, and date of next appointment.

**Table 2:** Questionnaires, instrumental and laboratory exams, and assessment of patient status used to evaluate the patient during the virtual visit

Activity	Exam/test	Means of evaluation
Self-evaluation questionnaires	• Pain	• NRS pain
	• Spasticity	• NRS spasticity
	• Depression	• HADS
	• Fatigue	• FSS
Follow-up exams	• Laboratory tests	
	• MRI	
Physical functioning	• Self-ambulation test	• TUG test
	• Equilibrium (eyes open and closed)	• Bohannon scale standing balance
	• Arms rigid and palms turned up • Resistance to force (upper limbs)	• Patient remains standing with palms facing upward
	• Resistance to force (lower limbs)	• 5XSST
	• Coordination test	• Patient touches nose with index finger of both hands • Pinching test and draw a shape test
	• General dialog	• Reply to physician questions
Dialog with patient	• Language	• Evaluate fluidity
	• Gestures	• Evaluate gestures
	• Verbal and visual memory	
	• Attention and concentration	
	• Reply to patient questions	
	• Awareness of disease	
	• Indications for management	
Administrative tasks	• Request for new exams	
	• Compile report	
	• Set date for next follow-up visit	

*NRS*, Numerical Rating Scale; *HADS*, Hospital Anxiety and Depression Scale; *FSS*, Fatigue Severity Scale; *BRBNT*, Brief Repeatable Battery of Neuropsychological Tests; *TUG*, Timed Up and Go; *5XSST*, 5X Sit-to-Stand Test

### Preliminary evaluation of the virtual visit

The program was started in December 2019 and has been operational since March 2020. As of October 2020, 25 virtual visits have been carried out. All these visits were carried out via Skype. The patient’s caregiver was present at all visits, and played an active role. All the tests proposed in the protocol were carried out at all visits, with the exception of the Pinching test and Drawing test, which were completed in 44% of virtual visits. Moreover, in 20% of cases, the neurologist held that the virtual visit was not sufficient to provide adequate information and the patient’s status and a follow-up in-person clinical visit was recommended. The average duration of the virtual visit was 24 min, while that of the pre-visit and post-visit were about 15 min each.

Physicians rated the virtual visit using the ad hoc questionnaire, scoring items from 1–10 (1 lowest, 10 highest). Overall satisfaction was considered high (mean score of 8.0 ± 0.5) and there were no technical problems (mean score of 9.4 ± 0.6). The patient was not perceived to have any problems with the visit itself and was able to communicate effectively (mean scores of 8.7 ± 0.6 and 9.6 ± 0.6, respectively), regardless of degree of disability. In agreeing with the statement that communication with the patient was not very different from in-person interaction, physicians rated this with a score of 6.5 ± 0.8. The physical activity tests during the virtual visit were considered sufficient to understand the clinical conditions of the patient (mean score of 6.7 ± 0.8), and were carried out without problems and considered adequate (each but one scored 8 at least).

Patient experience was also evaluated through the SUS. Overall, MS patients favorably rated the virtual visit with a mean SUS of 96.6 ± 6.1. Finally, the question for the patient “Would you recommend this system to another patient?” assessed by VAS obtained a mean score of 9.6 ± 0.7.

## Discussion

Considering the protocol and kits developed, we assessed the potential benefits of virtual follow-up of patients with MS (Table 3). For the patient, benefits were seen in terms of simplification of the logistics involved in the visit, while minimizing the risk of contagion with possibly reduced direct and indirect costs. For clinicians, a virtual follow-up visit allows the patient to be under continuous care and be efficiently managed, albeit at a distance. The protocol was also seen to have benefits for the healthcare structure by increasing the volume of patients under care with potentially increased cost savings.

**Table 3:** Potential benefits of virtual follow-up visits of patients with MS using the protocol devised

Benefit	Patient	Clinic	Healthcare structure
Simplification of logistics	•		
Minimize risk of contagion	•	•	
Continuity in care	•	•	•
Streamline patient visits		•	
Use of innovative solutions for management	•	•	•
Increase in volume of patients seen			•
Reinforcement of physician–patient contact		•	
Reduction in direct and indirect costs	•		•

While the present protocol for monitoring patients with MS was drafted before the ongoing COVID-19 pandemic, the use of telemedicine has become of pressing concern. In fact, recent guidance from the Italian Ministry of Health recommends the adoption of telemedicine when feasible [20]. The use of telemedicine must obviously be tailored to the individual condition and diagnostic and follow-up exams needed. The Virtual Visit Assessment (ViVA) structured virtual management protocol described herein is an important step in that direction. The ViVA protocol describes all the steps and exams to adopt that can be easily adopted to the needs of the individual center and patient.

The battery of tests that are part of the present protocol is not dissimilar than what has been proposed by others [18, 21]. In addition, it is flexible since the user can choose the type of virtual technology to be adopted based on what is most appropriate for the center and patient. Overall, the protocol developed herein was favorable assessed by both clinicians and patients, who said that they would propose and recommend the virtual visit to other patients. Of note, however, the virtual visit was not deemed adequate in 20% of cases in which a new relapse was suspected; thus, the patient was referred for an in-clinic visit. This is in line with previous reports on telemedicine in MS patients. For example, D’Haeseleer et al. reported that in 20 patients, 15% of virtual visits were not successful [18]. That a proportion of virtual visits can be expected: while a virtual visit may be satisfactory in the majority of patients, in others, and especially those with disease worsening or relapse, more accurate evaluation is needed.

In our evaluation, the patient’s caregiver was present at all virtual visits, and their presence is especially important when carrying out cognitive assessment. This is also a critical aspect in light of the current COVID-19 pandemic, where for the most part patients cannot be accompanied by caregivers in hospital, at least in Italy. Patients may also feel that the risk of infection is thus drastically lowered by a virtual visit. In this regard, a digital triage tool has recently been published that may help to identify patients with MS who are at high risk of COVID-19 infection [22].

A recent review on the use of telemedicine in patients with MS analyzed 28 studies in over 3200 patients. All considered, it was concluded that telemedicine is associated with benefits in terms of reducing the burden on the healthcare system and costs, and is appreciated by both patients and physicians [16]. Other experiences with telemedicine in patients with neurological conditions have also been reported that demonstrate its feasibility [23, 24]. Despite these benefits, it was noted that the optimal strategy for implementation is still lacking [16]. With the present structured protocol, we have attempted to fill that gap.

A virtual visit may additionally have the potential to further empower patients with MS. In Italy, while about one-third of patients with MS prefer a passive role, the vast majority (61%) desire a collaborative role [25]. Shared decision-making can form the basis of optimal patient care, and the ideal model of communication for patients with MS [26]. In this regard, empowering patients with MS is a central step in shared decision-making [26]. We found that care providers appeared to be satisfied with the alternative delivery of care using telemedicine.

Among the limitations of the study, only a small number of teleconsultations have been carried out in our center to date. Our currently limited experience certainly does not claim to provide scientifically reliable data, above all because the sample of patients we have tested so far is distorted by selection bias: it is likely that the clinician was led to propose ViVA to patients he knew to be technologically capable, and thus, these patients participated in the project more readily. Thus, more data are needed to better evaluate the effectiveness of ViVA. However, the main objective of the present publication was to present our structured protocol as it is our intention to implement the protocol on a broader scale. This will provide the opportunity to validate the protocol and assess its real-world efficacy. In this regard, cost analyses would also be of interest as has been performed in only a small number of studies.

As mentioned, the protocol was drafted prior to the ongoing COVID-19 pandemic, and it is clear that application of a telemedicine protocol, as per national guidance, has obvious advantages in terms of freeing structural and human resources during a time of profound crisis. Indeed, clinical practice and management of patients with MS has changed greatly in the past year [27]. In this regard, it would be worthwhile to further explore and expand upon the use of telerehabilitation [28–30].

There is controversy that COVID-19 has forever changed management of MS, with debate that teleconsultation might replace most clinical visits [17, 21, 31–34]. In favor of in-home televideo visits for follow-up in MS patients, such visits are feasible, rated favorable by both patients and clinicians, and are cost effective. Despite the value of a video neurological assessment compared to the in-clinic neurological assessment has not yet been provided, it is reasonable to say that most of neurological examination’s components can be easily assessed also via telemedicine. Televideo visits can be conducted with several instruments (such as computers with cameras, tablets, or smartphones), which are widely available. On the other hand, some patients (and physicians) might see a screen as a physical barrier, and the involvement of multiple neurological systems resulting in a combination of symptoms is impossible to overcome in the absence of in-person support. Moreover, inequalities such as access to a high-speed Internet connection and the availability of technologically valid devices can exist, and persons with MS and high disability levels might suffer from difficulties in using devices that are suitable for high-quality teleconsultations.

Notwithstanding these critical issues, our preliminary experience indicates the virtual protocol is more than satisfactory for many patients, and was highly rated by both patients and physicians. Telemedicine must be considered an additional tool for the future, while taking into account that neurological examinations and a direct health care professional-patient relationship still remain the basis of management of persons with MS, and therefore, being care is needed when deciding which visits should be in person and which remote. It will be of fundamental importance to maintain a balance between the need to limit current risks and inconveniences and the need to manage the disease in the best way.

## Supplementary Information

ESM 1 (DOCX 49 kb)

## Data Availability

Not applicable.
