# Supplementary material for: Description and preliminary experience with Virtual Visit Assessment (ViVA) during the COVID-19 pandemic, a structured virtual management protocol for patients with multiple sclerosis
Source: Neurol Sci. 2021 Jun 15;43(2):1207–14. doi: 10.1007/s10072-021-05371-3 (PMC8205205; doi:10.1007/s10072-021-05371-3)
Supplement: Supplementary file 1 — (DOCX 49 kb) [file 10072_2021_5371_MOESM1_ESM.docx]

**QUESTIONNAIRE FOR EVALUATION OF THE VIRTUAL VISIT SERVICE**

*(The original questionnaire was provided in Italian and translated for the readers’ convenience)*

Dear Doctor, with reference to the last "Virtual Visit" carried out, we ask you for a few minutes of your time to complete the following questionnaire. Rate each question on a scale from 1 (absolutely disagree) to 10 (absolutely agree).

## General information

1. Virtual visit date ________

2. Age range of the patient (18-35, 35-55, 56-64, >65)

3. Presence of the caregiver (Yes/No) Active role during the visit (Yes/No)

## Timing evaluation

4. Duration of the virtual visit

Pre-visit (min) __ Visit (min) __ Post-visit (min) __

5. The scheduled connection time was respected (Yes/No)

6. The overall duration of the interaction (connection, visit, closure) was adequate

| absolutely disagree | 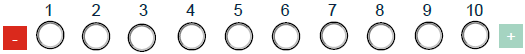 | absolutely agree |
| --- | --- | --- |

## Evaluation of the virtual interaction

7. I had connection problems during the call (Yes/No)

8. I had difficulties/problems using the technological solution chosen

| absolutely disagree | 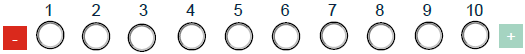 | absolutely agree |
| --- | --- | --- |

9. I had no particular problems in interaction during the visit

| absolutely disagree | 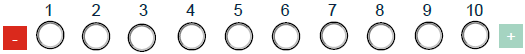 | absolutely agree |
| --- | --- | --- |

10. The patient was able to communicate effectively during the virtual visit

| absolutely disagree | 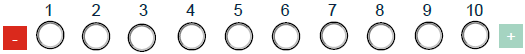 | absolutely agree |
| --- | --- | --- |

11. Communication with the patient through videoconferencing was not very different from interaction in person

| absolutely disagree | 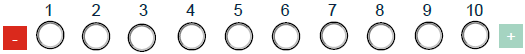 | absolutely agree |
| --- | --- | --- |

12. I do not think remote interaction was an obstacle to evaluation of the patient's clinical condition

| absolutely disagree | 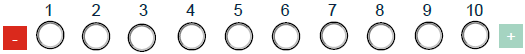 | absolutely agree |
| --- | --- | --- |

## Evaluation of carried out activities

13. The documents received by the patient before the visit (questionnaires, reports, etc.) were sufficient to understand the patient's condition

| absolutely disagree | 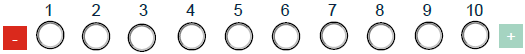 | absolutely agree |
| --- | --- | --- |

14. The patient's physical activity was carried out without problems

| absolutely disagree | 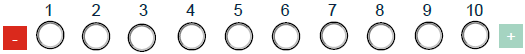 | absolutely agree |
| --- | --- | --- |

15. The tests performed during the visit were adequate

| absolutely disagree | 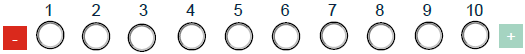 | absolutely agree |
| --- | --- | --- |

## Final evaluation

16. Overall evaluation of the virtual visit

| absolutely disagree | 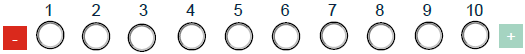 | absolutely agree |
| --- | --- | --- |

17. I had to refer the patient to an in-person clinical visit (Yes/No)

18. I want to propose other visits to the patient in virtual mode (Yes/No)

19. Notes
